# Atezolizumab in combination with intrathecal chemotherapy and radiation for treatment of isolated cerebral nervous system relapse in a patient with extranodal NK/T cell lymphoma: a case report

**DOI:** 10.1186/s13256-021-02740-6

**Published:** 2021-03-05

**Authors:** Amanda E. Lipsitt, Jaclyn Y. Hung, Anne-Marie Langevin

**Affiliations:** Department of Pediatrics Hematology-Oncology, University of Texas Health San Antonio, 7703 Floyd Curl Drive, Mail Code 7810, San Antonio, TX 78229 USA

**Keywords:** NK/T cell lymphoma, Non-Hodgkin’s lymphoma, Epstein-Barr virus, Immune checkpoint inhibitor, Atezolizumab, CNS relapse

## Abstract

**Background:**

Extranodal NK/T cell lymphoma (ENKTL) is an aggressive form of Epstein-Barr virus (EBV)-associated non-Hodgkin’s lymphoma which historically has a poor prognosis. When relapse occurs, particularly in the cerebral nervous system (CNS), survival is rare. The immune checkpoint pathway family of proteins is highly expressed in many human tumors, especially in EBV-related malignancies. To the best of our knowledge, there are no reports of immune checkpoint inhibitors used either alone or in combination for the treatment of ENTKL CNS relapse, yet there are promising results in metastatic CNS involvement of other malignancies.

**Case presentation:**

This is the case of a 29-year-old Hispanic male with ENKTL who was treated at first relapse with 24 doses of the programmed death-ligand 1 (PD-L1) immune checkpoint inhibitor, atezolizumab, over a 17-month period. He remained in remission for 18 months until he experienced an isolated CNS relapse and on-going evidence of chronic EBV infection. Salvage therapy was provided as a combination of triple intrathecal (TIT) chemotherapy, radiation, and atezolizumab. He continues on maintenance atezolizumab and remains alive 1-year post CNS relapse.

**Conclusions:**

The results from this case suggest that atezolizumab should be considered as part of the treatment regimen for relapsed ENKTL. They also demonstrate the benefit of using atezolizumab in combination with TIT chemotherapy and radiation as a viable treatment option for ENKTL CNS relapse and indicate that atezolizumab is an option for long-term maintenance therapy for patients with ENKTL.

## Background

Extranodal NK/T cell lymphoma (ENKTL) is an aggressive form of non-Hodgkin’s lymphoma that is difficult to treat and, with relapse, historically has a poor prognosis. The exact pathogenesis of ENKTL remains unknown, although it has been shown to be associated with Epstein–Barr virus (EBV) infection of tumor cells. A higher prevalence in the Asian and Hispanic populations coinciding with a rise in incidence in the USA is reported [1].

At presentation, ENKTL commonly causes localized destruction involving midline structures, such as the nose, sinuses, or palate; however, other areas of the body can also be affected, such as the gastrointestinal tract, skin, and testis [2]. Cerebral nervous system (CNS) involvement is uncommon, but has been reported in up to 17% of patients, although the rate tends to be dependent on the stage and histology of the tumor [3–7]. ENKTL is usually both chemotherapy and radiotherapy sensitive, but the prognosis remains poor for patients who relapse, especially those with CNS disease. Proposed treatments for these patients include intrathecal (IT) or high-dose methotrexate-based regimens [3, 8, 9].

Programmed death-ligand 1 (PD-L1), belonging to the immune checkpoint pathway family of proteins, is highly expressed in many human tumors, but especially in EBV-related malignancies. Multiple studies have demonstrated a particularly high expression of PD-L1 in ENKTL due to the EBV-infected T-cells [4, 10–12]. Therefore, immune checkpoint inhibitors can be a potential therapy for malignancies such as ENKTL which is related to EBV infection.

The PD-L1 immune checkpoint inhibitor, atezolizumab, shows promise as a treatment alternative in this devastating disease in the following case. Atezolizumab is a humanized immunoglobulin (IgG1) monoclonal antibody that selectively binds to PD-L1 and blocks its interaction with the programmed death-1 (PD-1) receptor and B7.1 (CD80), thus potentially activating an anticancer immune response. The U.S. Food and Drug Administration (FDA) has approved atezolizumab for the treatment of various cancer types, such as lung cancer, bladder cancer, and breast cancer [13].

Recently, other PD-1 inhibitors, such as pembrolizumab and nivolumab, both of which have a similar mechanism to atezolizumab, have emerged as successful treatments for multiple refractory, relapsed, and advanced ENKTL [14]. Pembrolizumab and nivolumab are now recommended as treatment choices for relapsed/refractory ENKTL per National Comprehensive Cancer Network (NCCN) guidelines last updated in 2018 [15].

The following is a report of a patient with relapsed ENKTL who achieved a second remission with atezolizumab and then was re-treated in combination with triple intrathecal (TIT) chemotherapy for isolated CNS relapse. Our aim in presenting this case report is to demonstrate the successful use of atezolizumab in the initial relapse, but specifically to highlight its potential role in the treatment of CNS relapse.

## Case presentation

**Fig. 1:**
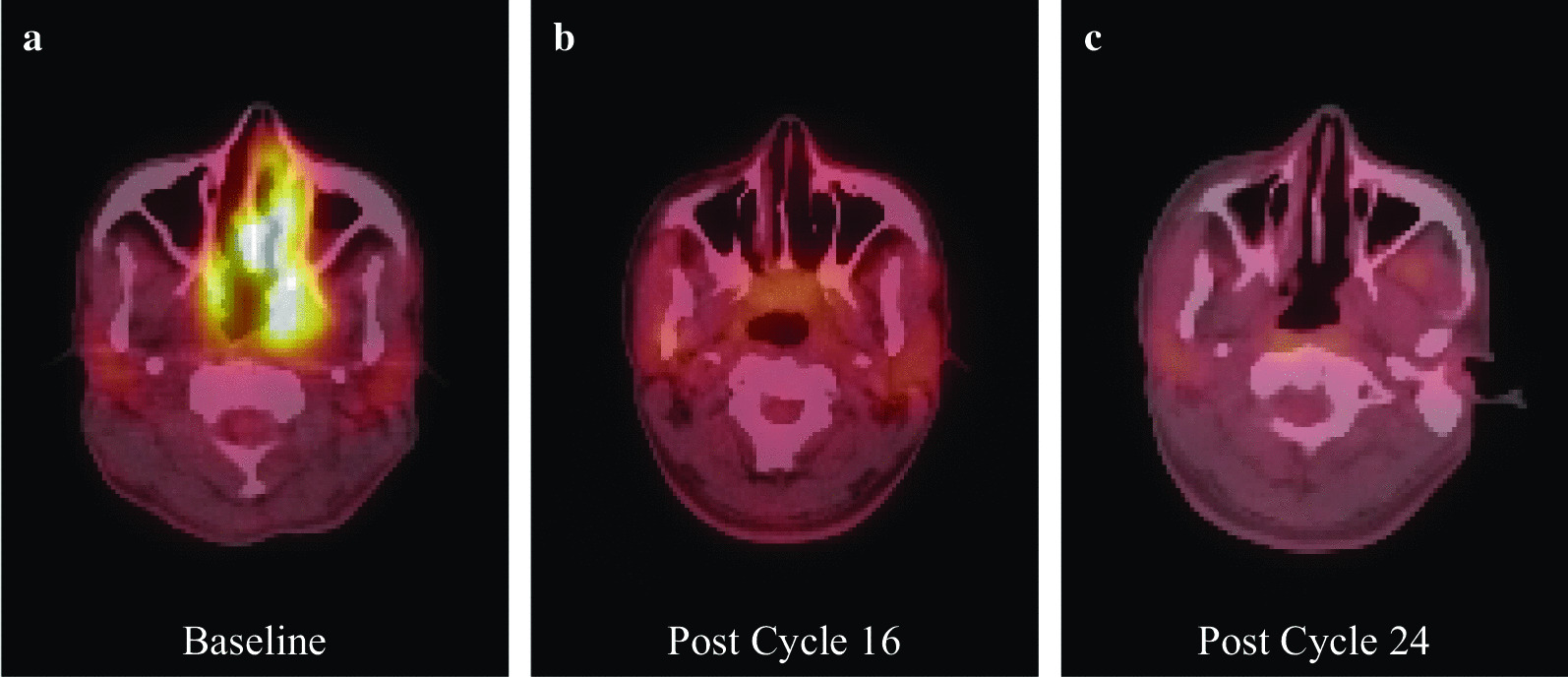
Positron emission tomography-computed tomography (PET-CT) scan at diagnosis of first relapse and after atezolizumab treatment. **a** Avid left cervical lymphadenopathy and nasopharynx, oropharynx, and soft palate at baseline. **b** After 16 doses of 1200 mg intravenous infusion of atezolizumab every 3 weeks, showing complete response of the target lesion and overall partial response. **c** After 24 doses of atezolizumab, the avid region was biopsied and confirmed by pathology to be negative for lymphoma

We report the case of a 29-year old Hispanic male with ENKTL with the first relapse in the nasopharynx and then an isolated relapse in the CNS. At initial diagnosis in 2012, at age 21 years, he presented with disease in the caecum, hypopharynx, and bone marrow. He was treated with the SMILE protocol (dexamethasone, methotrexate, ifosfamide, l-asparaginase, and etoposide) for 3 cycles, followed by autologous stem cell transplant with the BEAM regimen (carmustine, etoposide, cytarabine, and melphalan). He relapsed 4 years later, with presentation of left hemifacial pain, left otorrhea, fever, night sweats, fatigue, difficulty breathing through his nose, loss of smell, and loss of appetite. At this time, a positron emission tomography-computed tomography scan (PET-CT) revealed he had left cervical lymphadenopathy and nasopharynx, oropharynx, and soft palate avid (Fig. 1).

The patient was then enrolled in a clinical trial (ClinicalTrials.gov Identifier: NCT02541604) in August 2016 to receive 1200 mg (maximum dose) intravenous (IV) infusion therapy with atezolizumab every 3 weeks. Side effects were monitored according to the protocol. The target and non-target lesions were assessed by PET-CT after every two courses and evaluated by the Response Evaluation Criteria in Solid Tumors v1.1 (RECIST).

**Fig. 2:**
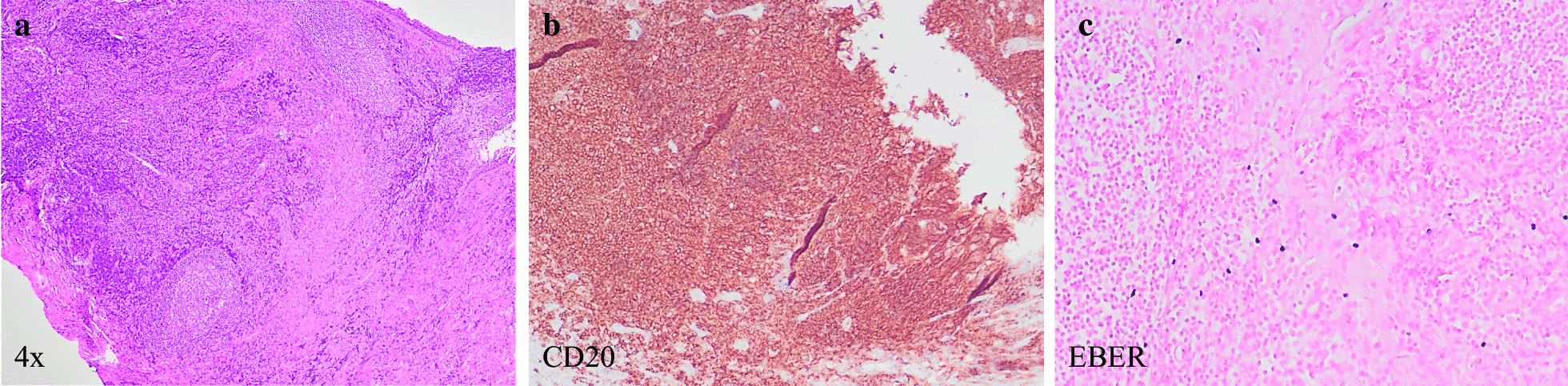
Biopsies of the PET-CT avid regions indicated scattered Epstein–Barr encoding region (*EBER*)-positive cells without evidence of lymphoma. **a** Hematoxylin and eosin (H&E) staining of sinonasal epithelium with underlying dense chronic inflammatory infiltrate, increased vasculature, thickened basement membrane, and increased goblet cells. Germinal center formation was present, with polarization and tingible body macrophages. Lymphoplasmacytic infiltrates were present in the interstitium. There were no areas of necrosis or mass lesions appreciated. **b** B cells predominately found in germinal centers were highlighted by CD20 positivity. **c** Increased scattered EBER–in situ hybridization-positive cells were seen as predominately small and stained. (×4 magnification)

The patient received his first dose of atezolizumab in September 2016 and his last dose in January 2018, for a total of 24 doses. At the start of treatment his EBV status was according to PCR analysis. Side effects were minimal without any Grade 3 or Grade 4 toxicity. He experienced striking clinical improvement within 3 weeks of receiving the first dose of atezolizumab. He reported increased appetite, weight gain, increased energy, disappearance of night sweats, no fever, and no missed days of work. At cycle 3, he was jogging approximately 3 miles per day about 5 days a week. At the end of treatment, the drainage from his left ear had almost completely disappeared. After completion of course 16, complete response (CR) of the target lesion and overall partial response (PR) were documented by PET-CT (Fig. 2). After course 24, biopsies of the PET-positive areas in the nasopharynx and tonsils were taken to assess the pathologic response. Biopsies showed scattered Epstein–Barr encoding region (EBER)-positive cells without evidence of lymphoma. The decision was then taken to stop the administration of atezolizumab due to sustained clinical response.

**Fig. 3:**
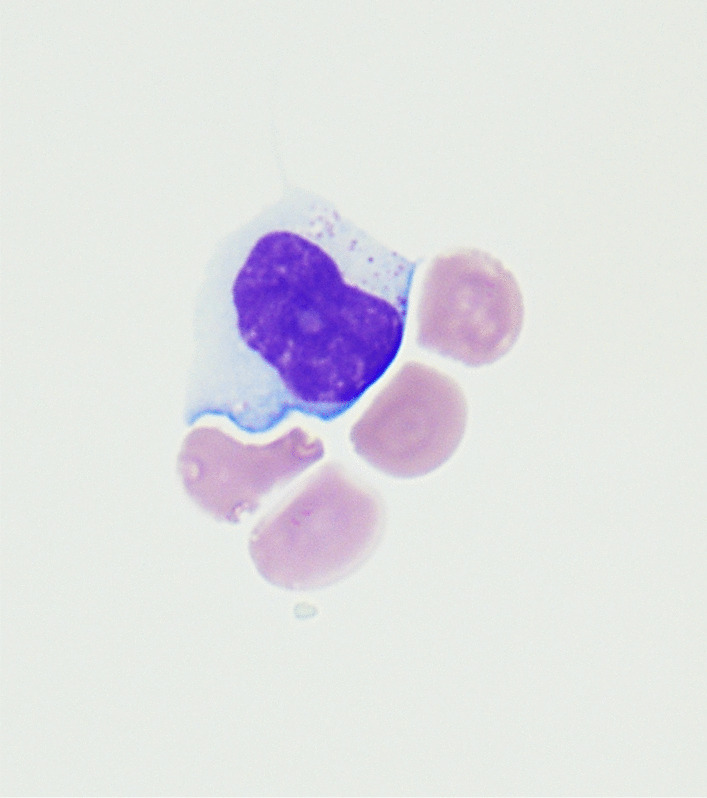
Cytospin preparation of the cerebrospinal fluid (CSF) at second relapse. Medium- to large-sized atypical lymphocyte with irregular and indented nuclear contours, slightly more open chromatin, and azurophilic cytoplasmic granules are present (H&E stain, × 100).

**Fig. 4:**
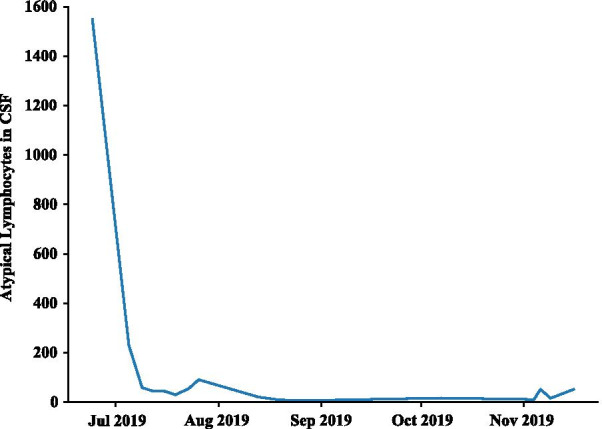
Atypical lymphocytes in the CSF during treatment with atezolizumab and triple intrathecal (TIT) chemotherapy following second relapse. At the patient’s second relapse, atypical lymphocytes were elevated at 1550. With atezolizumab infusion and TIT chemotherapy, the atypical lymphocyte count dropped significantly and remained very low over the course of the treatment

The patient remained disease free for approximately 18 months off treatment. During follow-up he developed diabetes and had an increasing number of EBV copies, as determined by PCR, despite antiviral therapy. He was assessed for EBV-targeted therapy but did not meet the criteria. In June 2019, he developed a headache and nuchal rigidity. The work-up determined that he had an isolated CNS relapse and on-going evidence of chronic EBV infection with the EBV detectable in blood and cerebrospinal fluid (CSF) (Figs. 3, 4); no additional sites of disease were identified. TIT chemotherapy consisting of methotrexate, hydrocortisone, and cytarabine was started and given twice a week, then weekly, and finally every 3 weeks. After 12 courses of TIT chemotherapy, the CSF still had disease and the plasma EBV load was still elevated; thus atezolizumab was added to the treatment regimen. Craniospinal radiation 3000 cGy to the brain and 2400 cGy to spine was started when it became evident that there was still EBER+ cells in the CSF. Following radiation, his EBV viral load was greatly diminished, but was still detectable albeit not quantifiable. Remarkably, the patient remains clinically well almost 1 year post CNS relapse while continuing on atezolizumab infusions.

## Discussion and conclusions

According to NCCN guidelines, a clinical trial is the preferred treatment option for patients with relapsed ENKTL. In the absence of a clinical trial, PD-1 inhibitors or a stem cell transplant, if the patient is eligible, are suitable alternatives [15]. Our patient previously had an autologous stem cell transplant, as this was the standard of care at the time of his initial diagnosis. In this case, allogeneic transplant was considered in the setting of both relapses. As described above, our patient had a high EBV viral load at the time of his second relapse. A recent study by Jeong et al. indicates that in patients with ENKTL and EBV present in the blood, progression-free survival after allogeneic transplant trends towards a poorer outcome [16]. In addition, there is no consensus on the use of stem cell transplant in the relapse setting [15, 17].

In our patient, the use of a checkpoint inhibitor administered in the context of a clinical trial was the more viable and individualized option since a clinical trial was available at the time of his first relapse. Furthermore, given a combination of psychosocial factors, including lack of funding, the decision was made to proceed with the atezolizumab clinical trial at first relapse. At time of his CNS relapse, the same agent was made available through the Medication Assistance Program (MAP). Allogeneic stem cell transplant was again considered but was rejected as an option since the use of checkpoint inhibitors prior to transplant predisposes patients to develop hyperacute graft-versus-host disease and increases the transplantation-related mortality [15, 17].

Although atezolizumab is FDA approved and used in the treatment of other cancer types, it has not been documented in the treatment of relapsed ENKTL with or without CNS involvement [13]. Our case demonstrates the successful use of atezolizumab at first and second relapse. This case also indicates a viable treatment option for those patients with CNS involvement when given in conjunction with TIT chemotherapy and radiation therapy.

The median overall survival for patients with ENKTL CNS disease is 3.8 months. Another study reported that patients with peripheral T-cell lymphoma relapse have a median overall survival of 1.5 months [3, 7]. Remarkably, our patient has greatly surpassed the median survival.

Studies have shown that pembrolizumab, a PD-1 inhibitor, is successful in treating patients with ENKTL relapse. In a study by Kwong et al. of seven patients treated with a median of seven cycles of pembrolizumab, patients with higher PD-L1 expression fared better. Interestingly, five of the patients were still in CR at the end of the 6-month follow-up period [14]. Similarly, Li et al. also reported a positive outcome in four of seven patients treated with a median of four cycles of pembrolizumab [18].

A small number of patients with ENKTL with CNS involvement have been followed to determine specific disease patterns and outcome. Determinants of a higher risk of CNS involvement include Ann Arbor stage III/IV, NK/T Cell Lymphoma Prognostic Index (NKPI) score of III or IV, location of extra-upper aerodigestive tract, and lymph node involvement [3, 4]. Our patient exhibited most of the risk factors identified for the development of CNS involvement.

Involvement of the CNS in ENKTL historically has a very poor prognosis with no standard treatment identified. A retrospective study by Nevel et al. described the outcomes of patients with ENKTL CNS disease. All patients received methotrexate (MTX) either systemically or intrathecally. Additional treatments varied greatly and included radiation, high-dose systemic chemotherapy, other IT chemotherapy, or autologous stem cell transplant [3]. Many patients were noted to succumb to infection and toxicity due to CNS-targeted treatment [3]. Of interest, our patient experienced few toxicities to atezolizumab alone or in combination with TIT.

There are currently no reports on the use of immune checkpoint inhibitors in patients with ENKTL CNS disease. However, there are promising results in other disease types with CNS involvement. Pembrolizumab has been shown to be effective against solid tumor metastasis of melanoma and non-small cell lung cancer, with a response rate ranging from 20 to 30% [19]. Similar results for CNS metastasis of melanoma and renal cell carcinoma are also reported for nivolumab [20, 21]. Interestingly, nivolumab is observed to be an effective treatment in rare extranodal large B-cell lymphomas of primary CNS lymphoma and CNS recurrence of primary testicular lymphoma [22].

EBV status is important when considering treatment with PD-L1 inhibitors since there is increased expression of PD-L1 in EBV-related cancers. A correlation between increased viral load in the cancer cells and cancer progression has been documented [23]. Of note, after completion of the atezolizumab course for the first relapse, our patient had an increased level of EBV viral load despite treatment with antiviral therapy. However, upon restarting atezolizumab as adjunct therapy to TIT and radiation therapy, the EBV viral load declined significantly.

Overall, there is no consensus on CNS prophylaxis in ENKTL, but NCCN guidelines recommend IT chemotherapy for adult T-cell leukemia/lymphoma, which tends to have a higher rate of relapse [7]. In their retrospective review of risk factors for CNS involvement in lymphoma, Kim et al. conclude that patients with NKPI groups III and IV might benefit from prophylaxis; however, this conclusion was based on 12 patients identified with CNS disease [24]. One question raised by our case is whether the continued use of atezolizumab as maintenance therapy could have prevented the CNS relapse. Also, our case indicates that patients with ENKTL with CNS relapse may benefit from treatment with immune checkpoint inhibitors. Taken together, our case and observations from other studies suggest that further investigation is warranted on the use of immune checkpoint inhibitors in maintenance therapy and for treatment of CNS disease.

## Data Availability

Data sharing is not applicable to this article as no datasets were generated or analyzed during the current study

## Abbreviations

CNS: Cerebral nervous system
CR: Complete response
EBER: Epstein–Barr encoding region
EBV: Epstein-Barr virus
ENKTL: Extranodal NK/T cell lymphoma
FDA: Food and Drug Administration
IT: Intrathecal
NCCN: National Comprehensive Cancer Network
NKPI: NK/T Cell Lymphoma Prognostic Index
PD-1: Programmed death-1
PD-L1: Programmed death-ligand 1
PET-CT: Positron emission tomography-computed tomography
PR: Partial response
PCTL: Peripheral T-cell lymphoma
RECIST: Response Evaluation Criteria in Solid Tumors
TIT: Triple intrathecal

## References

[1] Al-Hamadani M, Habermann TM, Cerhan JR, Macon WR, Maurer MJ, Go RS. Non-Hodgkin lymphoma subtype distribution, geodemographic patterns, and survival in the US: a longitudinal analysis of the National Cancer Data Base from 1998 to 2011. Am J Hematol. 2015;90(9):790–5. 10.1002/ajh.24086 26096944

[2] Tse E, Kwong YL. The diagnosis and management of NK/T-cell lymphomas. J Hematol Oncol. 2017;10(1):85. 10.1186/s13045-017-0452-9 PMC5391564 28410601

[3] Nevel KS, Pentsova E, Daras M. Clinical presentation, treatment, and outcomes of patients with central nervous system involvement in extranodal natural killer/T-cell lymphoma. Leuk Lymphoma. 2019;60(7):1677–84. 10.1080/10428194.2018.1551541 PMC7580871 30648449

[4] Kim TM, Park YH, Lee SY, Kim JH, Kim DW, Im SA, et al. Local tumor invasiveness is more predictive of survival than International Prognostic Index in stage I(E)/II(E) extranodal NK/T-cell lymphoma, nasal type. Blood. 2005;106(12):3785–90. 10.1182/blood-2005-05-2056 16109779

[5] Gurion R, Mehta N, Migliacci JC, Zelenetz A, Moskowitz A, Lunning M, et al. Central nervous system involvement in T-cell lymphoma: a single center experience. Acta Oncol. 2016;55(5):561–6. 10.3109/0284186X.2015.1118656 PMC5376067 27046135

[6] Ellin F, Landström J, Jerkeman M, Relander T. Central nervous system relapse in peripheral T-cell lymphomas: a Swedish Lymphoma Registry study. Blood. 2015;126(1):36–41. 10.1182/blood-2014-12-616961 25957393

[7] Chihara D, Fanale MA, Miranda RN, Noorani M, Westin JR, Nastoupil LJ, et al. The risk of central nervous system relapses in patients with peripheral T-cell lymphoma. PLoS One. 2018;13(3):e0191461. 10.1371/journal.pone.0191461 PMC5851529 29538376

[8] Bokstein F, Lossos A, Lossos IS, Siegal T. Central nervous system relapse of systemic non-Hodgkin’s lymphoma: results of treatment based on high-dose methotrexate combination chemotherapy. Leuk Lymphoma. 2002;43(3):587–93. 10.1080/10428190290012092 12002763

[9] El-Galaly TC, Cheah CY, Bendtsen MD, Nowakowski GS, Kansara R, Savage KJ, et al. Treatment strategies, outcomes and prognostic factors in 291 patients with secondary CNS involvement by diffuse large B-cell lymphoma. Eur J Cancer. 2018;93:57–68. 10.1016/j.ejca.2018.01.073 PMC5869165 29477102

[10] Chen BJ, Chapuy B, Ouyang J, Sun HH, Roemer MG, Xu ML, et al. PD-L1 expression is characteristic of a subset of aggressive B-cell lymphomas and virus-associated malignancies. Clin Cancer Res. 2013;19(13):3462–73. 10.1158/1078-0432.CCR-13-0855 PMC4102335 23674495

[11] Bi XW, Wang H, Zhang WW, Wang JH, Liu WJ, Xia ZJ, et al. PD-L1 is upregulated by EBV-driven LMP1 through NF-κB pathway and correlates with poor prognosis in natural killer/T-cell lymphoma. J Hematol Oncol. 2016;9(1):109. 10.1186/s13045-016-0341-7 PMC5064887 27737703

[12] Jo JC, Kim M, Choi Y, Kim HJ, Kim JE, Chae SW, et al. Expression of programmed cell death 1 and programmed cell death ligand 1 in extranodal NK/T-cell lymphoma, nasal type. Ann Hematol. 2017;96(1):25–31. 10.1007/s00277-016-2818-4 27696202

[13] U.S. Food and Drug Administration (FDA). Drugs@FDA: FDA-approved drugs. https://www.accessdata.fda.gov/scripts/cder/daf/index.cfm?event=overview.process&ApplNo=761041. Accessed 24 Apr 2020.

[14] Kwong YL, Chan TSY, Tan D, Kim SJ, Poon LM, Mow B, et al. PD1 blockade with pembrolizumab is highly effective in relapsed or refractory NK/T-cell lymphoma failing l-asparaginase. Blood. 2017;129(17):2437–42. 10.1182/blood-2016-12-756841 28188133

[15] Network NCCN. T-cell lymphoma (version 1.2020). 2020. https://www.nccn.org/professionals/physician_gls/pdf/t-cell_blocks.pdf. Accessed 18 May 2020.

[16] Jeong SH, Song HN, Park JS, Yang DH, Koh Y, Yoon SS, et al. Allogeneic stem cell transplantation for patients with natural killer/T cell lymphoid malignancy: a multicenter analysis comparing upfront and salvage transplantation. Biol Blood Marrow Transpl. 2018;24(12):2471–8. 10.1016/j.bbmt.2018.07.034 30064012

[17] Yamaguchi M, Suzuki R, Oguchi M. Advances in the treatment of extranodal NK/T-cell lymphoma, nasal type. Blood. 2018;131(23):2528–40. 10.1182/blood-2017-12-791418 29602763

[18] Li X, Cheng Y, Zhang M, Yan J, Li L, Fu X, et al. Activity of pembrolizumab in relapsed/refractory NK/T-cell lymphoma. J Hematol Oncol. 2018;11(1):15. 10.1186/s13045-018-0559-7 PMC5793390 29386072

[19] Goldberg SB, Gettinger SN, Mahajan A, Chiang AC, Herbst RS, Sznol M, et al. Pembrolizumab for patients with melanoma or non-small-cell lung cancer and untreated brain metastases: early analysis of a non-randomised, open-label, phase 2 trial. Lancet Oncol. 2016;17(7):976–83. 10.1016/S1470-2045(16)30053-5 PMC5526047 27267608

[20] Long GV, Atkinson V, Lo S, Sandhu S, Guminski AD, Brown MP, et al. Combination nivolumab and ipilimumab or nivolumab alone in melanoma brain metastases: a multicentre randomised phase 2 study. Lancet Oncol. 2018;19(5):672–81. 10.1016/S1470-2045(18)30139-6 29602646

[21] De Giorgi U, Cartenì G, Giannarelli D, Basso U, Galli L, Cortesi E, et al. Safety and efficacy of nivolumab for metastatic renal cell carcinoma: real-world results from an expanded access programme. BJU Int. 2019;123(1):98–105. 10.1111/bju.14461 29956884

[22] Nayak L, Iwamoto FM, LaCasce A, Mukundan S, Roemer MGM, Chapuy B, et al. PD-1 blockade with nivolumab in relapsed/refractory primary central nervous system and testicular lymphoma. Blood. 2017;129(23):3071–3. 10.1182/blood-2017-01-764209 PMC5766844 28356247

[23] Nakayama A, Abe H, Kunita A, Saito R, Kanda T, Yamashita H, et al. Viral loads correlate with upregulation of PD-L1 and worse patient prognosis in Epstein-Barr Virus-associated gastric carcinoma. PLoS One. 2019;14(1):e0211358. 10.1371/journal.pone.0211358 PMC6350976 30695048

[24] Kim SJ, Oh SY, Hong JY, Chang MH, Lee DH, Huh J, et al. When do we need central nervous system prophylaxis in patients with extranodal NK/T-cell lymphoma, nasal type? Ann Oncol. 2010;21(5):1058–63. 10.1093/annonc/mdp412 19850636

